# Production and application of polyclonal and monoclonal antibodies against *Spiroplasma eriocheiris*

**DOI:** 10.1038/srep17871

**Published:** 2015-12-07

**Authors:** Ying Zhang, Haixun Bao, Fengqin Miao, Yaqin Peng, Yuqing Shen, Wei Gu, Qingguo Meng, Wen Wang, Jianqiong Zhang

**Affiliations:** 1 Key Laboratory of Developmental Genes and Human Disease, Ministry of Education, Department of Microbiology and Immunology, Medical School, Southeast University, 87 Dingjiaqiao Road, Nanjing 210009, China; 2 Jiangsu Key Laboratory for Biodiversity & Biotechnology and Jiangsu Key Laboratory for Aquatic Crustacean Diseases, College of Life Sciences, Nanjing Normal University, 1 Wenyuan Road, Nanjing 210023, China

## Abstract

A new species of spiroplasma, *Spiroplasma eriocheiris* (*S. eriocheiris*), was identified as a lethal pathogen of tremor disease (TD) in Chinese mitten crab recently. In order to acquire appropriate biological and diagnostic tools for characterizing this newly discovered pathogen, 5 monoclonal antibodies (mAbs) and a polyclonal antibody (pAb) against *S. eriocheiris* were produced. Among the mAbs, 6F5, 7C8 and 12H5 lead to the deformation of *S. eriocheiris.* A peptide sequence, YMRDMQSGLPRY was identified as a mimic motif of MreB that is the cell shape determining protein of *S. eriocheiris* interacting with 3 mAbs. Furthermore, a double antibody sandwich enzyme-linked immunosorbent assay (DAS-ELISA) for detection of *S. eriocheiris* was established using the mAb and pAb we prepared. It detected as low as 0.1 μg/mL of *S. eriocheiris*. No cross-reaction was observed with three other common bacteria (*Pseudomonas aeruginosa*, *Escherichia coli*, and *Bacillus subtilis*) and the hemolymph samples of healthy *Eriocheir sinensis*. Collectively, our results indicated that the mAbs and pAb we prepared could be used in the analysis of *S. eriocheiris* membrane proteins mimotope and development of a diagnostic kit for *S. eriocheiris* infections.

The Chinese mitten crab, *Eriocheir sinensis*, is an economically important species cultured in China. Tremor disease (TD) is one of the most serious epizootics of the crab that adversely affect harvests in recent years. In 2002, it was the first time that a pathogenic spiroplasma in a crustacean was detected; and subsequently it was identified as a causative agent of TD in 2004^1^. In 2010, it was given the name *Spiroplasma eriocheiris*^1^. *S. eriocheiris* infects not only the crab but also three other freshwater crustaceans: pacific white shrimp (*Litopenaeus vannamei*), crayfish (*Procambarus clarkii*) and prawn (*Macrobrachium rosenbergii* and *Macrobrachium nipponense*), leading to a massive death^2^.

Spiroplasmas are wall-less, helical, motile prokaryotes, which were most often found in association with insects and ticks. At a much lower frequency, they have also been isolated from plants^3^. Recent isolations from crustaceans are beginning to widen our understanding of the host range. As a member of cell-wall-free mollicutes, its membrane proteins are of primary importance in adherence, invasion, and interaction with the host, and they may also display structural, transport, or enzymatic functions^4^. To date, three types of *S. eriocheiris* surface proteins, SLP25 (Spiralin-like protein), SLP31 and ALP41 (adhesin-like protein), have been cloned and expressed^4^,^5^,^6^. In our previous study, we generated and characterized the first mAb (6H7) against *S. eriocheiris*, and using this mAb, we identified a motif of ALP41^7^. The analysis of more mimotopes of *S. eriocheiris* membrane proteins and potential roles of them will be helpful in further research on characterization of *S. eriocheiris* and its pathogenesis.

There is no specific treatment or vaccine for TD, so rapid and accurate diagnosis during the early course is essential to control this disease. *S. eriocheiris* infection can be detected by several molecular and/or immunological techniques^8^. However, most of them require special equipment and expensive reagents except the enzyme-linked immunosorbent assay (ELISA) method, which has been used for many years as a field diagnostics. An indirect ELISA using pAb prepared for the rapid detection of *S. eriocheiris* was developed, but it is time-consuming and the sensitivity and specificity needs to be improved^8^.

The main objective of our study was to generate and characterize more mAbs and pAb against *S. eriocheiris*. These antibodies were used to analyse mimotopes of its membrane proteins and to develop DAS-ELISA, enhancing the ability to detect and confirm suspected cases of *S. eriocheiris* infection. This in turn may reduce TD mortality and direct strategies for controlling infection.

## Results

### Characterization of the pAb and mAbs 5C11, 5D9, 6F5, 12H5, 7C8

Whole-cell *S. eriocheiris* and *S. eriocheiris* cells broken by ultrasonic homogenizer were used separately as Ag to produce mAbs. After the fusion of the host spleen cells with the myeloma cells, we found that the ratio of fusion from the former type of antigen was about 80%, while that from the latter was 70%. Indirect ELISA was done to screen for the hybridoma cells that could secrete mAbs capable of binding to *S. eriocheiris*. The cells that have strong ELISA reactivities with *S. eriocheiris* were subsequently subjected to cloning procedures. Five clones (5C11, 5D9, 6F5, 12H5 and 7C8) with higher titer, affinity, and good cell growth status were finally obtained for further characterization.

**Figure 1:**
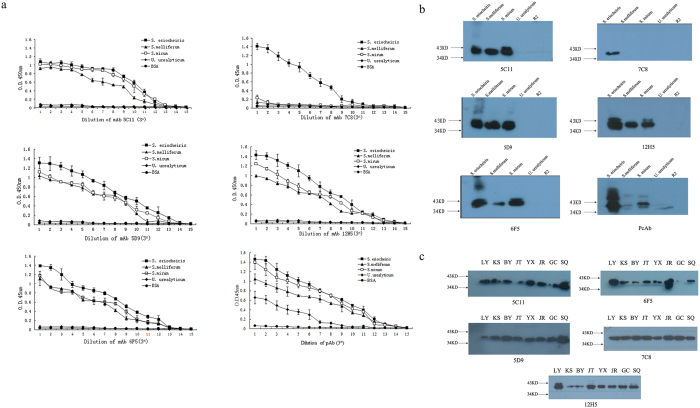
(**a**) Reactions of the mAbs 5C11, 5D9, 6F5, 12H5, 7C8 and pAb to different species of *Spiroplasmas* or *U. urealyticum* by indirect ELISA assay. The wells were coated with 300 ng whole cell lysates of *S. eriocheiris* (■), *S. melliferum* (▲), *S. mirum* (◻) and *U. urealyticum* (♦). BSA (●) was used as control. The mAbs and pAb initially adjusted to 1 mg mL^−1^ was tested in fifteen three-fold dilutions. First dilution 3^1^ (n = 1) and last 3^15^ (n = 15). Optical density was measured at 450 nm. The experiment was repeated three times, and the results are presented as mean ± SD. (**b**) Reactions of the mAbs and pAb with the whole cell lysates of *S. eriocheiris*, *S. melliferum*, *S. mirum*, *U. urealyticum*, and culture medium of the Spiroplasmas (R2) used as control. (**c**) Reactions of the mAbs with *S. eriocheiris* strains from eight areas by Western blot assay. *S. eriocheiris* strains of different areas (Lane 1: Liyang; Lane 2: Kunshan; Lane 3: Baoying; Lane 4: Jintan; Lane 5: Yixing; Lane 6: Jurong; Lane 7: Gaochun; Lane 8: Suqian).

The titers (expressed as the reciprocal of the ascites or serum dilution) of the mAbs reached 3^11^–3^14^, and that of pAb was 3^14^ as determined by indirect ELISA. Specificity analyses of the mAbs and pAb were done by indirect ELISA and Western blotting. The results of indirect ELISA assay showed that 7C8 reacted with *S. eriocheiris* when it was diluted from 1:3^1^ to 1:3^11^, but did not cross-react with *S. melliferum*, *S. mirum* or *U. urealyticum* (Fig. 1a). Moreover, the other four mAbs reacted with *S. eriocheiris*, *S. melliferum* and *S. mirum*, but not with *U. urealyticum* (Fig. 1a). The pAb reacted with all of the 4 members of Mollicutes, while not with the negative control (Fig. 1a). These results suggested that mAb 7C8 recognized a *S. eriocheiris* specific epitope, while the other four mAbs recognized an epitope common to all of the 3 spiroplasmas. The results were further confirmed by Western blot assay, which revealed that mAb 7C8 was capable of identifying the protein band (about 40 kDa) and is in good accordance with those of the other four mAbs (Fig.1b).

The light-chain isotypes of the 5 mAbs (5C11, 5D9, 6F5, 12H5 and 7C8) were κ, while the heavy-chain isotypes were not the same by detection using mouse mAb isotyping test kit. Affinity constant (K_aff_) of the mAbs was measured by indirect ELISA. The results are summarized in Table 1. As shown, these mAbs exhibited higher affinity for *S. eriocheiris*. The K_aff_ ranged between 7.04 × 10^6^ and 6.21 × 10^8^.

Above-mentioned data showed that a strong specific response could be elicited by the mAb 7C8, and it is relatively specific for the other four mAbs. In order to verify if the motifs recognized by the mAbs were shared by the strains from different areas in Jiangsu province, *S. eriocheiris* strains isolated from *E. sinensis* of TD in 8 different areas in Jiangsu province were detected with the 5 mAbs by Western blot analysis. The results showed that the mAbs reacted with all of the *S. eriocheiris* strains collected from the above areas (Liyang, Kunshan, Baoying, Jintan, Yixing, Jurong, Ggaochun and Suqian), implying that the binding epitopes of these mAbs were conserved among these strains (Fig. 1c).

### Effects of the mAbs on the biological characteristics of *S. eriocheiris*

**Figure 2:**
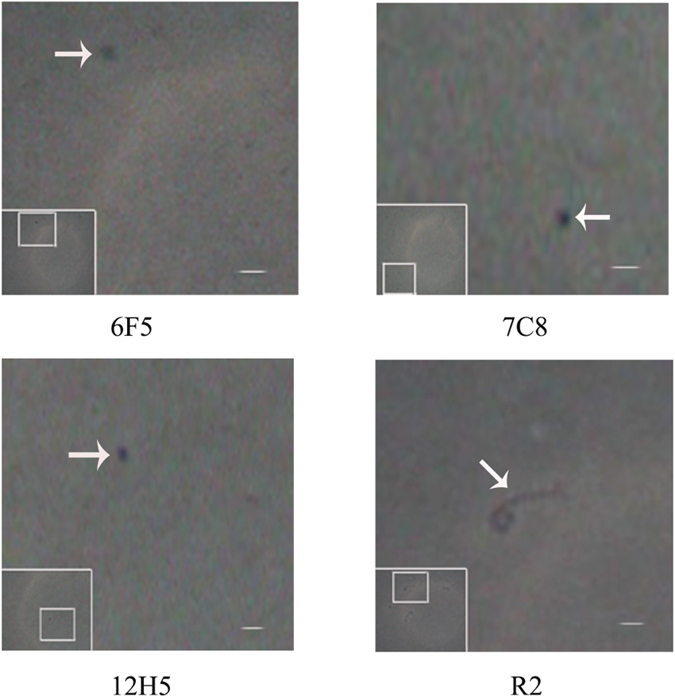
Effects of mAbs 6F5, 7C8, 12H5 on the shapes of *S. eriocheiris*. Oil immersion lens micrographs of samples of *S. eriocheiris* cultures in the logarithmic phase of growth added with 6F5, 7C8, 12H5 or R2, showing round (6F5, 7C8, 12H5) and helical (R2) structures. Arrows indicate the intact and deformed cells on images.

A dark field microscope was used to observe the samples for helicity. Most *S. eriocheiris* in the presence of mAb 5D9, 5C11 or absence of any mAb exhibited initial helicity (Fig. 2 R2). While mAb 6F5, 7C8 or 12H5 deformed 20%–30% of *S. eriocheiris*, showing round structures (Fig. 2 6F5, 7C8, 12H5).

On R2 agar, *S. eriocheiris* produced small yellow colonies after 17–25 days of incubation at 30 °C, and there were not any red zones of inhibition of *S. eriocheiris* growth surrounding the disks saturated with the mAbs or R2 medium. This means the mAbs we tested did not inhibit the growth of *S. eriocheiris*.

There was simultaneously yellowing in *S. eriocheiris* suspension added with various dilutions of Abs compared with the control. This means the mAbs we tested did not inhibit the metabolism of *S. eriocheiris*.

### Screening of phage display peptide libraries and characterization of recombinant phages

Analysis of peptide segments displayed on the surface of phage is a useful method to identify partners of the known proteins^9^,^10^. To investigate the binding motif of mAbs 6F5, 7C8 or 12H5 which deformed *S. eriocheiris*, the Ph.D.-12 libraries were screened with the purified mAbs. The yield rate increased significantly from the first to the fourth round of biopanning (1.63 × 10^−6^, 8.63 × 10^−4^, 3.16 × 10^−3^, and 2.29 × 10^−2^, respectively), suggesting that high-affinity-binding peptides to the mAbs were significantly enriched during the screening procedure.

**Figure 3:**
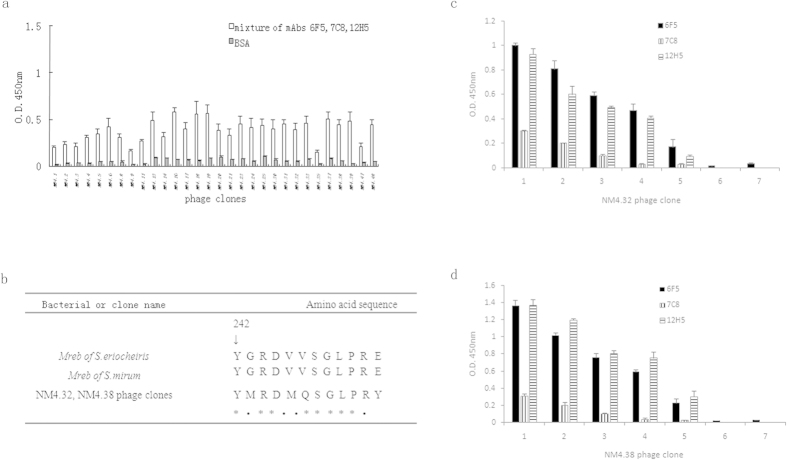
(**a**) Binding of different phages to the mAbs 6F5, 7C8 and 12H5 detected by indirect ELISA. (**b**) Amino acid sequence comparisons between pep-NM4.32, NM4.38 and the related region of cell shape determining protein (Mreb) of *S. eriocheiris* and *S. mirum* using CLUSTAL W ver.3.1. The mark, ‘asterisk’ or ‘dot’, placed in the amino acid sequence indicates identical or different residues, respectively. (**c**,**d**) 6F5, 7C8 and 12H5’s binding to NM4.32 or NM4.38 phage clone. ELISA was performed using plates coated with 6F5, 7C8 or 12H5, in the presence of NM4.32 or NM4.38 phage clone of serial dilutions. The binding of phage clone to the mAbs was detected by a HRP-conjugated anti-M13 mAb. BSA or unrelated mAb was also coated in the presence of NM4.32 or NM4.38 phage clone (2.2 × 10^11^ virions/well) as a negative control.

Among the 48 individual clones selected from biopanning, 30 clones were successfully sequenced. The deduced amino acid sequences of the corresponding inserts revealed 9 sequences. Interestingly, we found all these 30 clones showed higher signals in the phage ELISA binding assay (Fig. 3a). Among them, the amino acid sequence of pep-NM4.32 and NM4.38 was highly homologous to 242–253 amino acids of cell shape determining protein (Mreb1 and MreB4) from *S. eriocheiris* and *S. mirum* (Fig. 3b). These two clones were chosen for further analysis. Both NM4.32 and NM4.38 exhibited a significant, dose-dependent binding to 6F5, 7C8 and 12H5, even with 0.9 × 10^9^ virions per well, while not binding to the irrelevant murine mAb (Fig. 3c,d). These results demonstrated that the residues (YMRDMQSGLPRY) contained a binding site to mAbs 6F5, 7C8 and 12H5.

### Establishment of double antibodies sandwich ELISA (DAS-ELISA) for rapid detection of *S. eriocheiris*

#### Antigen preparation

Whole-cell *S. eriocheiris* was used in the following establishment of DAS-ELISA. 100 μL *S. eriocheiris* of 32 μg/mL was used as the positive control, and PBS as the negative control according to the indirect ELISA previously established.

### Determination of the best working concentration

A checkerboard titration was applied to investigate the best working concentration of the coating polyclonal Ab and horseradish peroxidase conjugated *S. eriocheiris* specific mAb 6H7 (HRP-6H7). Results showed that the relatively high P/N value can be obtained when the concentration of the coating pAb was 1 μg/mL or 0.5 μg/mL (Table 2). To avoid excessive use of the pAb, the latter concentration was used in the following experiments. For HRP-6H7, the maximum OD value (about 0.5-0.6) was obtained when the concentration of HRP-6H7 was 0.2 μg/mL, and the OD value decreased with the reduction of its concentration (Table 2). So 0.5 μg/mL pAb and higher concentration of HRP-6H7 (2 μg/mL, 0.4 μg/mL and 0.2 μg/mL) were used to investigate the best working concentration of HRP-6H7. As shown in Table 3, when the concentration of HRP-6H7 was 2 μg/mL, the maximum P/N (15.4) value can be obtained, the OD value of the positive control was nearly 1, and that of the negative control was less than 0.1. From the above results, the best working concentration was 0.5 μg/mL for PcAb, and 2 μg/mL for enzyme labelled mAb.

### Determination of sensitivity, specificity and repeatability of the DAS-ELISA

**Figure 4:**
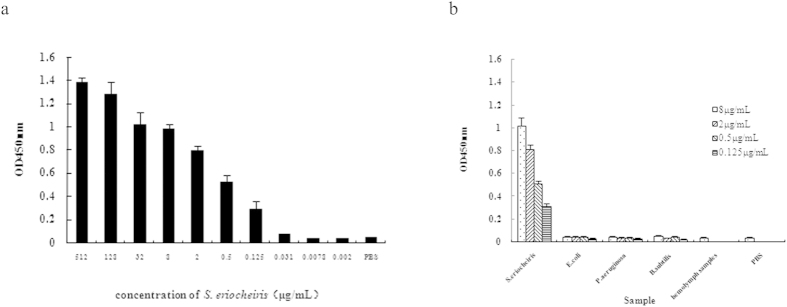
(**a**) Analytical sensitivity of the DAS-ELISA. The abscissa was the concentration of *S. eriocheiris* and ordinal coordinates was OD values. The original antigen protein content was 512 μg/mL. The lowest concentration that *S. eriocheiris* could be detected by the DAS-ELISA was 0.125 μg/mL. (**b**) Specificity of the DAS-ELISA. Hemolymph samples obtained from healthy, uninfected crabs and PBS were used as a negative control.

To determine the sensitivity of the DAS-ELISA, the concentration of *S. eriocheiris* was examined using 4-fold serial dilutions (512 μg/mL, 128 μg/mL, 32 μg/mL, 8 μg/mL, 2 μg/mL, 0.5 μg/mL, 0.125 μg/mL, 0.031 μg/mL, 0.0078 μg/mL, 0.002 μg/mL). As depicted in Fig. 4a, a minimal detection limit of 0.125 μg/mL (OD450nm = 0.289) was obtained according to the cut-off value (P/N > 2.1).

Three different bacterial species (*B. subtilis*, *P. aeruginosa*, *E. coli*) which are commonly found in aquaculture were employed to identify the cross-reactivity of the DAS-ELISA. No significant cross-reactivity was detected according to the results. The OD450 values of the 3 bacterial species were similar to that of hemolymph samples obtained from healthy, uninfected crabs and PBS, which indicated the DAS-ELISA method has a high specificity for *S. eriocheiris* (Fig. 4b).

Three different concentrations (8 μg/mL, 2 μg/mL and 0.5 μg/mL) of *S. eriocheiris* were used to calculate the intra- and inter-plate coefficient of variation (CV). CV of the intra-plate and inter-plate were both lower than 10% (Table 4 and Table 5), which is within the expected range of ELISA. The intra- and inter-plate coefficient of variations were used to validate the repeatability of the DAS-ELISA. These results indicated that the DAS-ELISA we developed offered high repeatability.

## Discussion

Spiroplasma diseases are most often found in association with insects and plants^3^. However, one species within this group, *S. eriocheiris*, was isolated from several freshwater aquatic crustaceans including *E. sinensis*, *L. vannamei*, *P. clarkii*, *M. rosenbergii* and *M. nipponense*^1^,^2^.

This spiroplasma is of additional interest because of its well-established role as a pathogen of freshwater aquatic crustaceans, and is of significant economic importance^1^. As a new isolated and important pathogen, elucidation of the molecular characteristics and development of a rapid, convenient detection method are urgently needed.

Antibodies are important tools for the study of the characteristics, pathogenesis, rapid detection methods, prevention and treatment of pathogens^11^. A polyclonal antibody against *S. eriocheiris* was developed soon after the spiroplasma was identified. It was used in the indirect ELISA to detect *S. eriocheiris* in crab hemolymph^8^. It is known that mAbs have significant advantages over polyclonal counterparts in the epitope analysis and differentiation of pathogens because of their binding with high specificity and high affinity to a target molecule. Developing mAbs should be very important because mAbs specific to *S. eriocheiris* will be a useful tool in the study of antigenic epitopes of *S. eriocheiris* proteins and epidemiological survey of TD. To date, only one mAb 6H7 has been produced^7^. Catastrophic economic losses caused by outbreaks of TD enabled the production of more mAbs and pAbs described in the present study. In this study we prepared 5 mAbs and a pAb against *S. eriocheiris* by immunizing mice or rabbits with *S. eriocheiris*.

Among the five mAbs, 7C8 reacted specifically to *S. eriocheiris* as shown by ELISA and Western blotting, but not to other microbes we tested. It suggests that the epitope detected by this mAb is highly specific to the newly defined spiroplasma, just like the first mAb 6H7 we produced. To date, many specific mAbs have been used in the establishment of immunological assays for the detection of pathogenic microbial antigens^12^,^13^,^14^. These 2 mAbs can therefore be used to distinguish *S. eriocheiris* from other spiroplasmas.

Diagnosis of *S. eriocheiris* infection is essential for the treatment of tremor disease. Presently, some methods requiring special equipment and expensive reagents, such as light microscopy, electron microscopy and PCR-based molecular techniques, have been used for identification of *S. eriocheiris* from crustaceans and other aquatic animals^8^. ELISA is more suitable for field applications in aquaculture farms because of its simplicity, inexpensiveness, and reliability; and there have been a few reports of ELISA detection for spiroplasmas from plants and insects tissues^15^,^16^,^17^. Indirect ELISA was established using the polyclonal antibody against *S. eriocheiris*^8^. Among various ELISA formats, DAS-ELISA is most commonly used and time-saving in the detection of the pathogen. And a series of mAbs and pAb we produced also offered a wider choice of reagents for the establishment of this method. The surface proteins are the most appropriate target for the diagnosis of pathogen infection. We have two *S. eriocheiris* specific mAbs (7C8 and 6H7) which can be used to develop the DAS-ELISA. There are two reasons for why the latter was chosen as an agent for the establishment of the assay. Firstly, 6H7 had higher affinity to *S. eriocheiris* than 7C8. Secondly, one of our goals to develop mAbs against *S. eriocheiris* is to establish a convenient and efficient method to detect viable *S. eriocheiris* cells; therefore, the sensitivity and reliability of this method is critical to achieve this goal. MAb 6H7 proved to have high binding affinity to ALP41, which is an important and constitutional expressed surface protein of *S. eriocheiris*. To improve the sensitivity, pAb combined with more epitopes was coated to capture more relevant antigens. The minimal detection limit is lower than that of the indirect ELISA established previously, which indicated that DAS-ELISA may offer higher sensitivity. This seemed attractive in detecting subclinical infections. It is noteworthy that no background was observed in the hemolymph specimens which were examined by the protocol described herein. So DAS-ELISA was proven useful for detecting *S. eriocheiris* antigens in clinical samples.

In the cell-wall-free mollicutes, membrane molecules are of primary importance in adherence, invasion, shape determination, and interaction with the host immune system. And they may also display structural, transport, or enzymic functions^18^,^19^. MAbs 6F5, 7C8 and 12H5 we produced were able to deform 20% to 30% of *S. eriocheiris*. The 3 mAbs may interact with epitopes of shape determination-associated proteins or an epitope shared by various proteins functionally related to shape determination. Thus, we identified the binding motif of the mAbs via a phage display library (Ph.D.-12).

The sequence (YMRDMQSGLPRY) containing a binding site to the mAbs was highly homologous to amino acid sequence 242–253 of Mreb1 and MreB4 (cell shape determining protein) from *S. eriocheiris* and *S. mirum*. MreB is the bacterial homologue of eukaryotic actin. MreB genes are present only in bacterial species with rod-shaped, filamentous, or helical cells^20^. Its major function appears to be the control of cell shape, mainly by directing cell wall building blocks to their destination^21^. Since Spiroplasma lacks a cell wall, MreB’s function is different from that in *E. coli.* It has been identified in *S. citri* and *S. melliferum* which is a protein about 36 kDa^21^,^22^. And it is likely that it forms a membrane-associated helical ribbon, and then a highly ordered linear motor positioned on a defined helical line along the internal face of the cell’s membrane. There are five mreB homologs in the draft genome sequences of these two spiroplasmas, encoding MreB proteins with a molecular weight of 38 kDa (unpublished data). The functional roles of these MreB homologs remain to be investigated^23^. We assume that the mAbs deformed *S. eriocheiris* by binding the motifs of MreBs or other proteins with the same conformational epitope, triggering mechanical forces responsible for the loss of helicity. As a component of the cytoskeleton, MreB is firmly attached to the cytoplasmic face of the membrane. Although the studies suggested that the mAbs were likely interacting with MreB, how and where this interaction was occurring remained a mystery. It is possible that MreB may span the plasma membrane, leaving a small region exposed outside of the cell while another terminus is located on the cytosolic side under some conditions just like HSP72^24^,^25^,^26^.

*S. eriocheiris* and *S. mirum* are 98.8% identical at the nucleotide sequence level in MreB1, 97.9% in MreB4. The molecular basis of antigen-antibody interaction is that the CDR (complementarity-determining region) of Ab binds to the small region of a large antigen which is called the antigenic epitope. Studies of many Ag-Ab interfaces have revealed that the contact residues of antigenic epitope are often discontinuous in sequence but contiguous in space. That means the antibody recognizes the overall shape of an epitope rather than particular chemical residues. Ag-Ab reactions can show a high level of specificity. Ab is able to distinguish between small differences in the primary amino acid sequence of proteins, in addition to differences in charge, optical configuration and steric conformation. The single amino acid mutations in the protein, although fairly distant from the contact residues, appeared to induce subtle conformational changes leading to the changes of the interaction with antibody^27^,^28^. Therefore, it is proposed that few different amino acids of MreB1/4 at *S. eriocheiris* and *S. mirum* alter the partial conformation of the proteins, thereby forming common and specific motifs of MreB1/4 between *S. eriocheiris* and *S. mirum*. MAb 7C8 may bind *S. eriocheiris* specific motifs of MreB1/4, while mAbs 6F5 and 12H5 bind common ones of them, consistent with the results before (Fig. 1a,b). As the peptide displayed on the surface of the phage presents a primary conformation, 7C8 showed significantly lower signals in the phage ELISA binding assay than the other two mAbs (Fig. 3c,d).

In conclusion, we generated and characterized 5 mAbs directed against *S. eriocheiris*. The epitopes of *S. eriocheiris* detected by 7C8 are quite different from that by the other four mAbs; therefore, the mAbs may have different biological effects. In addition, these mAbs could offer new approaches for the study of *S. eriocheiris*. Used as ligands, they could enable the isolation and purification of *S. eriocheiris* proteins utilizing methods such as affinity chromatography or immunomagnetic separation. A specific motif (FQGINHYNQMER) of cell shape determining protein (Mreb) of *S. eriocheiris* was identified from a phage display peptide library by using mAbs 6F5, 7C8 or12H5 which deformed *S. eriocheiris* as baits. This motif and the 3 mAbs can be used as biological tools in further research on the role of MreB of *S. eriocheiris* in both the physiology and pathogenesis. As a result of our research, a DAS-ELISA for the detection of the pathogen was established, using rabbit anti-*S. eriocheiris* serum as a coating antibody, HRP-labelled 6H7 as the detecting antibody, and finally an optimized protocol was developed. It was sensitive and specific for *S. eriocheiris* detection, simple and time-saving to produce and perform compared to existing serological methods. Our results indicated that the DAS-ELISA is a suitable method for the diagnosis of *S. eriocheiris* infection and could be used in serological surveys to map out the prevalence of TD.

## Methods

### Ethics statement

All experimental protocols were approved by the ethics committee of the Medical School of Southeast University, Nanjing, Jiangsu. The methods were carried out in accordance with the approved guidelines. All animal experiments were conducted in accordance with the protocols evaluated and approved by Institutional Animal Care and Use Committee (IACUC) of the Medical School of Southeast University (approval ID: SYXK-2010.4987).

### Bacterial strains

*Spiroplasma mirum* was purchased from the American Type Culture Collection (ATCC 29335). *S. eriocheiris* was isolated from *E. sinensis* that were exhibiting the tremor disease using a previously described method^1^.

### Preparation of immunogen and production of mAbs

Whole-cell *S. eriocheiris* and *S. eriocheiris* cells broken by ultrasonic homogenizer were used as antigens^7^. MAbs were produced using the standard procedures described previously^7^,^29^,^30^. The ascites containing mAbs were collected and purified with protein A/G affinity columns (Millipore, USA). The concentration of the mAb was determined with the BCATM Protein Assay Kit (Thermo, USA). The established hybridoma cell lines were stored in liquid nitrogen for later use. The Ig isotype of the mAb was identified using the IsoStrip Mouse Monoclonal Antibody Isotyping Kit (Roche, Germany) according to the manufacturer’s instructions.

### Rabbit immunization and pAb preparation

The Poly Ab was generated by immunizing the New Zealand white rabbits with bacteria as previously described with some modification^31^. Briefly, rabbits were immunized with 6 × 10^8^ *S. eriocheiris* cells by hypodermic injection with equal volumes of Freund’s complete adjuvant. The immunization was repeated at days 21, 35, 49 and 63 after the first injection with Freund’s incomplete adjuvant. The animals were bled one week after the fifth injection and the sera were collected by centrifuging clotted blood at 2,000 g and stored at −20 °C. Preimmunization sera were obtained and used as a control. All antisera were heat-inactivated at 56 °C for 30 min and filtered through 450-nm membrane filters. The sera were purified with protein A/G affinity columns (Millipore, USA). The concentration of the pAb was determined with the BCATM Protein Assay Kit (Thermo, USA).

### Indirect ELISA

Indirect ELISA for screening anti-*S. eriocheiris* hybridoma, measuring titrations of *S. eriocheiris* antibodies in affinity-purified ascites or rabbit serum and testing the specificities of the Abs to other species of spiroplasmas or *Ureaplasma urealyticum* were done as we previously described^7^. The end point dilution method was used to determine the titer of antibodies by the indirect ELISA. Antibody titers were defined as the reciprocal of the highest ascites or serum dilution that produced an O.D value above the cutoff^7^. The experiment was repeated 3 times, and the results are presented as mean ± SD.

### The affinity constant (Kaff) of mAb for binding to *S. eriocheiris*

The affinity constant of the mAb was determined as we described before^7^,^32^.

### Western blot assay for testing the specificities of the affinity-purified mAbs or pAb to other species of spiroplasmas or Ureaplasma urealyticum

Standard procedures of Western blotting were used to determine the reactivity of the mAb or pAb to the whole cell lysates of *S. eriocheiris*, *S. melliferum*, *S. mirum* and *U. urealyticum* on a 12% gel^7^. The mAbs or pAb we prepared were used as the primary antibody and the HRP conjugated goat anti-mouse IgG Fc or anti-rabbit as the secondary antibody (Chemicon, Japan). The culture medium of spiroplasmas or pre-immunized rabbit serum was used as the negative control.

### Western blot assay for testing the reactivities of the mAbs to the *S. eriocheiris* strains from eight different areas in Jiangsu province

The procedure was similar to that described above.

### Effects of mAbs on the biological characeristics of *S. eriocheiris*

#### Deformation test

Deformation test was done according to the method reported before with slight modifications^33^. Briefly, 50 μl mAbs to be examined were added to the same volume of *S. eriocheiris* cultures in the logarithmic phase of growth. *S. eriocheiris* cultures were used as a control. The treated samples were incubated at room temperature for thirty minutes and then examined using dark-field microscopy. The control was read first.

### Growth inhibition test

Samples (0.1 mL) of *S. eriocheiris* cultures in the logarithmic phase of growth were plated onto plate agar surfaces consisting of sucrose and indicator-Phenol red. Thirty minutes later, the sterile disks saturated with mAbs were placed on the surface of the agar at the center of the dried inoculated plates. The plates were then incubated in 30 °C incubator. The sterile disk saturated with R2 medium was used as a negative control.

### Metabolism inhibition test

Two fold dilutions of Abs and tenfold dilutions of *S. eriocheiris* were made firstly. Two hundred μL of *S. eriocheiris* cultures were added to 50 μL mAbs to be examined, then mixed thoroughly. R2 medium, *S. eriocheiris* culture and Abs served as different controls. All tubes were placed in 30 °C incubator for 3 to 10 days. When the color of the medium control remained unchanged and *S. eriocheiris* suspension turned yellow, the highest dilution of Ab in which the metabolism of *S. eriocheiris* were inhibited was recorded.

### Screening the phage display library using the mAbs which have effects on the biological characeristics of *S. eriocheiris* as the target molecule and sequencing DNA

Ph.D.-12 Phage Display Peptide Library (New England Biolabs, USA) was used in this study. Four rounds of biopanning were undertaken according to the manufacturer’s instructions to investigate the binding motif of mAbs which have effects on the biological characeristics of *S. eriocheiris*. After the fourth round of panning, 48 individual blue plaques were picked from LB/IPTG/X-gal plates randomly. They were cloned and amplified. The inserted DNA was sequenced and translated into peptides. Homology searches and multiple sequence alignments were performed using DNA Star and Clustal W programs to determine the similarities between the consensus peptides of *S. eriocheiris* and *S. mirum*.

### Phage ELISA binding assay

To test the binding specificities of the selected phage clones, phage ELISA binding assay was performed by coating one row of ELISA plate with mAbs for each phage clone. The amounts of recombinant phages bound to the mAbs were determined using HRP-conjugated anti-M13 antibody. All phage clones were tested in triplicate, and the results are presented as mean ± SD.

### Establishment of double antibody sandwich ELISA

#### Preparation of antigens

Whole-cell *S. eriocheiris* were used as antigens in the DAS-ELISA. Other aquaculture-associated bacterial isolates (*Bacillus subtilis*, *Pseudomonas aeruginosa* and *Escherichia coli*,) were obtained by courtesy of Southeast University, Department of Pathogenic Biology and Immunology. Ultrasonic fragmentation of the agents were used as control antigens.

### Conjugation of mAb to horseradish peroxidase

Horseradish peroxidase was conjugated to the *S. eriocheiris* specific mAb 6H7 with Peroxidase Labeling Kit according to the manufacturer’s instructions (Roche Ltd).

### Development of DAS-ELISA

Microplates (MaxiSorp, Nunc) were coated with anti-*S. eriocheiris* rabbit serum of the optimized working concentration (in 0.05 M carbonate buffer, pH 9.6, 100 μL/well) overnight at 4 °C. A blocking buffer (PBS-T containing 2% rabbit serum, 200 μL/well) was added to the plate for 1 h at 37 °C followed by thrice washing with PBS-T. After the plate was washed as described above, the sample (diluted with rabbit serum containing PBS) was added and the plate was incubated at 37 °C for 1 h. *S. eriocheiris* of known concentrations (32 μg/mL, 100 μL/well) was used as positive control according to the previous study and PBS as negative control. Positive and negative controls were treated in the same way. After washing as before, horseradish peroxidase conjugated mAb 6H7 (HRP-6H7) was added at the optimized working concentration and incubated once more for 1 h at 37 °C. The plate was then treated with the tetramethylbenzidine substrate followed by washing 5 times. After incubation in the dark for approximately 10 min, a stopping solution (2 mol/l H_2_SO_4_, 50 μL/well) was added. The absorption was measured at 450 nm using a microplate reader. The experiment was repeated three times, and the results are presented by mean ± SD.

### Optimization of working concentration

To develop a highly sensitive and specific DAS-ELISA, assay conditions were optimized according to the checkerboard titration method^34^, including the concentration of coating anti-*S. eriocheiris* rabbit serum and HRP-6H7. The optimized working concentration of coating pAb and HRP-6H7 were determined via the ratio of the OD value of the positive to the value of the negative (P/N).

### Determination of sensitivity, specificity and repeatability of the DAS-ELISA

#### Sensitivity of the DAS-ELISA

Different concentrations of *S. eriocheiris* (512 μg/mL, 128 μg/mL, 32 μg/mL, 8 μg/mL, 2 μg/mL, 0.5 μg/mL, 0.125 μg/mL, 0.031 μg/mL, 0.0078 μg/mL, 0.002 μg/mL) were examined to determine the lowest concentration of antigen based on the working concentration of pAb and HRP-6H7. The values of P/N larger than 2.1 were considered as positive.

### Cross-reactivity Assessment of the DAS-ELISA

*B. subtilis*, *P. aeruginosa*, *E. coli* and hemolymph samples obtained from healthy, uninfected crabs and sterile PBS were tested against the DAS-ELISA we developed. *S. eriocheiris* were used as positive control and PBS as negative control.

### Repeatability of the DAS-ELISA

The DAS-ELISA we developed was used to assess *S. eriocheiris* of 3 different concentrations (8 μg/mL, 2 μg/mL and 0.5 μg/mL) with four microplates to calculate the variation. Each concentration of *S. eriocheiris* were tested in three replicates within any of the four microplates. The coefficients of variation (CV = SD/Mean × 100%) were calculated and the intra-plate variation and inter-plate variation were used to validate the repeatability of the DAS-ELISA^35^.

## Additional Information

**How to cite this article**: Zhang, Y. *et al.* Production and application of polyclonal and monoclonal antibodies against *Spiroplasma eriocheiris*. *Sci. Rep.* **5**, 17871; doi: 10.1038/srep17871 (2015).

## Figures and Tables

**Table 1:** Identification of subclasses, titers and affinity constants for mAbs.

Clones number	Isotype	Affinity constant (M^−1^)	Titre	Specificities
5C11	IgG1/ κ	1.37 × 10^7^	3^12^	*S. eriocheiris* + , *S. melliferum* + ,
				*S. mirum* + , *U. urealyticum - *
5D9	IgG1/ κ	7.04 × 10^6^	3^13^	*S. eriocheiris* + , *S. melliferum* + ,
				*S. mirum* + , *U. urealyticum - *
6F5	IgG1/ κ	6.21 × 10^8^	3^13^	*S. eriocheiris* + , *S. melliferum* + ,
				*S. mirum* + , *U. urealyticum - *
7C8	IgG2a/ κ	1.19 × 10^8^	3^11^	*S. eriocheiris* + , *S. melliferum*−,
				*S. mirum* + , *U. urealyticum - *
12H5	IgG1/ κ	1.66 × 10^8^	3^14^	*S. eriocheiris* + , *S. melliferum* + ,
				*S. mirum* + , *U. urealyticum - *


The mark ‘ + ’ or ‘−’ in specificities indicates reacted with or not reacted with.

**Table 2:** Determination for optimal concentration of the coating PcAb and the detecting HRP-McAb.

			concentration of the PcAb (μg/mL)						
			6	4	2	1	0.5	0.25	0.125
**concentration of HRP-6H7 (μg/mL)**	0.2	P	0.61 ± 0.014	0.548 ± 0.006	0.567 ± 0.005	0.541 ± 0.007	0.561 ± 0.01	0.541 ± 0.007	0.507 ± 0.007
		N	0.071 ± 0.007	0.058 ± 0.004	0.047 ± 0.005	0.036 ± 0.004	0.036 ± 0.04	0.038 ± 0.006	0.035 ± 0.003
		PN	8.59	9.45	12.1	15	15.6	14.2	14.5
	**0.1**	P	0.483 ± 0.008	0.445 ± 0.007	0.401 ± 0.006	0.395 ± 0.005	0.434 ± 0.06	0.409 ± 0.01	0.405 ± 0.005
		N	0.037 ± 0.003	0.031 ± 0.003	0.038 ± 0.004	0.018 ± 0.002	0.022 ± 0.006	0.023 ± 0.002	0.021 ± 0.008
		PN	13.1	14.4	10.6	21.9	19.7	17.8	19.3
	**0.067**	P	0.373 ± 0.011	0.339 ± 0.005	0.346 ± 0.002	0.359 ± 0.007	0.359 ± 0.004	0.342 ± 0.006	0.328 ± 0.006
		N	0.03 ± 0.005	0.029 ± 0.003	0.031 ± 0.003	0.03 ± 0.006	0.028 ± 0.005	0.031 ± 0.006	0.023 ± 0.009
		PN	12.4	11.7	11.2	12	12.8	11	14.3
	**0.05**	P	0.287 ± 0.004	0.281 ± 0.005	0.311 ± 0.003	0.317 ± 0.004	0.296 ± 0.008	0.285 ± 0.006	0.284 ± 0.013
		N	0.03 ± 0.003	0.027 ± 0.008	0.027 ± 0.003	0.023 ± 0.003	0.027 ± 0.005	0.019 ± 0.006	0.015 ± 0.005
		PN	9.6	10.4	11.5	13.8	10.9	15	19

“P”: the OD value of the positive control which was read at 450 nm.

“N”: the OD value of the negative control which was read at 450 nm.

“P/N”: the ratio of the OD value of the positive to the value of the negative.

**Table 3:** Determination for optimal concentration of the detecting HRP-McAb.

concentration of the PcAb (0.5 μg/mL)			
concentration of HRP-6H7(μg/mL)	2	P	0.998 ± 0.02
		N	0.065 ± 0.007
		P/N	15.4
	0.4	P	0.663 ± 0.05
		N	0.047 ± 0.004
		P/N	14.1
	0.2	P	0.458 ± 0.04
		N	0.052 ± 0.005
		P/N	8.8

“P”: the OD value of the positive control which was read at 450 nm.

“N”: the OD value of the negative control which was read at 450 nm.

“P/N”: the ratio of the OD value of the positive to the value of the negative.

**Table 4:** Intra-plate coefficient of variation of the DAS-ELISA for *S. eriocheiris*.

Concentration of S.eriocheiris (μg/mL)	No.		OD value		Within plate		
					Mean	S D	C V(%)
8	I	0.972	0.864	0.924	0.92	0.054	0.059
	II	0.875	0.936	0.952	0.921	0.041	0.044
	III	0.895	0.872	0.898	0.888	0.014	0.016
	IV	0.94	0.883	0.879	0.901	0.034	0.038
2	I	0.813	0.746	0.808	0.789	0.037	0.047
	II	0.825	0.698	0.791	0.771	0.066	0.085
	III	0.764	0.711	0.634	0.703	0.065	0.093
	IV	0.779	0.814	0.701	0.765	0.058	0.076
0.5	I	0.536	0.55	0.467	0.518	0.044	0.086
	II	0.584	0.574	0.506	0.555	0.042	0.077
	III	0.535	0.516	0.473	0.508	0.032	0.063
	IV	0.487	0.524	0.589	0.533	0.052	0.097

“S D”: Standard deviation.

“C V”: Coefficient of variation.

“No.”: Plate number.

**Table 5:** Inter-plate coefficient of variation of the DAS-ELISA for *S. eriocheiris*.

Concentration of S.eriocheiris(μg/mL)	Mean of the OD value of different plates				Between plates		
	I	II	III	IV	Mean	S D	C V(%)
8	0.92	0.9	0.888	0.901	0.908	0.016	0.017
2	0.789	0.8	0.703	0.765	0.757	0.037	0.049
0.5	0.518	0.6	0.508	0.533	0.528	0.02	0.039

“S D”: Standard deviation.

“C V”: Coefficient of variation.
